# DoseGAN: a generative adversarial network for synthetic dose prediction using attention-gated discrimination and generation

**DOI:** 10.1038/s41598-020-68062-7

**Published:** 2020-07-06

**Authors:** Vasant Kearney, Jason W. Chan, Tianqi Wang, Alan Perry, Martina Descovich, Olivier Morin, Sue S. Yom, Timothy D. Solberg

**Affiliations:** Department of Radiation Oncology, University of California, San Francisco, CA 94115 USA

**Keywords:** Radiotherapy, Prostate cancer, Biological physics

## Abstract

Deep learning algorithms have recently been developed that utilize patient anatomy and raw imaging information to predict radiation dose, as a means to increase treatment planning efficiency and improve radiotherapy plan quality. Current state-of-the-art techniques rely on convolutional neural networks (CNNs) that use pixel-to-pixel loss to update network parameters. However, stereotactic body radiotherapy (SBRT) dose is often heterogeneous, making it difficult to model using pixel-level loss. Generative adversarial networks (GANs) utilize adversarial learning that incorporates image-level loss and is better suited to learn from heterogeneous labels. However, GANs are difficult to train and rely on compromised architectures to facilitate convergence. This study suggests an attention-gated generative adversarial network (DoseGAN) to improve learning, increase model complexity, and reduce network redundancy by focusing on relevant anatomy. DoseGAN was compared to alternative state-of-the-art dose prediction algorithms using heterogeneity index, conformity index, and various dosimetric parameters. All algorithms were trained, validated, and tested using 141 prostate SBRT patients. DoseGAN was able to predict more realistic volumetric dosimetry compared to all other algorithms and achieved statistically significant improvement compared to all alternative algorithms for the V_100_ and V_120_ of the PTV, V_60_ of the rectum, and heterogeneity index.

## Introduction

Advanced treatment techniques such as intensity modulated radiation therapy (IMRT) and volumetrically modulated arc therapy (VMAT) have become standard of care for many treatment sites^1,2^. Creating clinically acceptable treatment plans using these advanced techniques requires extensive domain expertise and is exceedingly time consuming^3,4^. To reduce the burden on clinical resources, the development of automated treatment planning technologies has accelerated in recent years^5–10^.

Historically, automated treatment planning technologies relied on selecting handcrafted features, such as spatial relationships between planning volumes, overlapping volume histograms, planning volume shapes, planning volume and field intersections, field shapes, planning volume depths , and distance-to-target histograms (DTH)^11–14^. These techniques rely on machine learning algorithms such as gradient boosting, random forests, and support vector machines to find strong correlations between groups of weakly correlated predictive features^6,15–17^. Such techniques achieve good performance on inherently structured data, but tend to struggle if the problem does not easily reduce to a structured format. Because of this, deep learning approaches have emerged that predict dose using fully connected layers^18^. However, fully connected layers tend to not generalize well on highly dimensional data.

Convolutional neural networks (CNNs) have emerged to solve many image processing tasks^4,6,19–23^. Recently, encoder-decoder CNNs have been used to predict radiation dose from arbitrary patient anatomy. These methods rely on voxel-voxel or pixel-pixel loss to update network parameters, since the objective function needs to be differentiable^24^. Stylistic variations in human planner preferences make direct spatial loss functions prone to learning overly smooth dosimetric distributions. Additionally, stereotactic body radiation therapy (SBRT) and stereotactic radiation surgery (SRS) treatment modalities tend to produce random hotspots residing within the gross tumor volume (GTV)^25,26^. Since conventional CNNs learn to predict the most probable dose, they are not well suited to model SBRT or SRS dose distributions^20,27,28^.

Recently, generative adversarial networks have been used to facilitate realistic predictions, by training a secondary CNN to distinguish real from fake predictions^29–32^. The generator CNN aims to create realistic predictions that fool a discriminator CNN, which attempts to classify realism. The two networks are trained adversarially until a Nash equilibrium is reached, which is the minimax loss of the aggregate training protocol^33^. Since the two networks need to be trained in unison, the discriminator network is usually shallow with fewer parameters compared to stand-alone classification CNNs such as VGG-16, ResNet-151, or DenseNet-201 architectures^29^. However, conventional GANs rely on the discriminator’s ability to distinguish fake predictions from real predictions, so the overall performance is limited by the discriminator’s ability decipher realism^34^.

Attention gates have recently emerged to help networks highlight relevant anatomy and suppress irrelevant information by encouraging compatibility between the input, intermediate layers, and output function of the network^35,36^. Additive self-attention gates have been proposed to encourage parsimonious feature propagation throughout a network^37–39^. Spatial self-attention allows networks to selectively emphasis portions of the intermediate convolutional layers as opposed to indiscriminately propagating information using conventional raster scanning.

This study suggests a novel attention-gated generative adversarial network (DoseGAN) as a superior alternative to current state-of-the-art dose prediction networks. DoseGAN offers deeper and more efficient discrimination, while simultaneously being efficient enough to train in unison with the generator network.

## Methods and materials

### Attention gated generation and discrimination

DoseGAN utilizes attention-gated generation and discrimination networks that selectively propagate information through a gating mechanism. The attention gates enable the networks to highlight relevant input features and help suppress redundant information propagation through the network. The gating mechanism also helps encourage compatibility between the output function and the extracted intermediate local feature vectors in each network^35,36^. DoseGAN utilizes additive self-attention gates to modulate multi-scale level feature response propagation throughout each network^37–39^.

**Figure 1:**
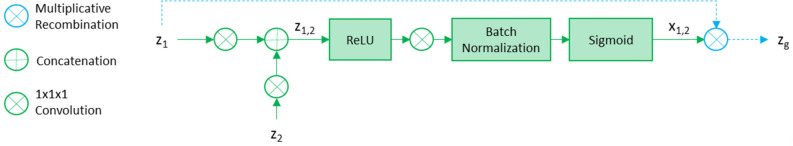
The attention gating mechanism is shown for the propagation signal z_1_, gating signal z_2_, and final gated output signal z_g_ for the discriminator and generator networks.

The attention-gating mechanism applies a 1 × 1 × 1 convolutional kernel to a propagation signal (z_1_) and a gating signal (z_2_). Signals z_1_ and z_2_ are added together and the combined activations (z_1,2_) are ReLU activated before being passed through a 1 × 1 × 1 convolutional kernel. The output is batch normalized and sigmoidally activated to form x_1,2_. The final gated output signal (z_g_) is formed by multiplying z_1_ by x_1,2_. Figure 1 depicts the attention gating mechanism used in the discriminator and generator networks.

**Figure 2:**
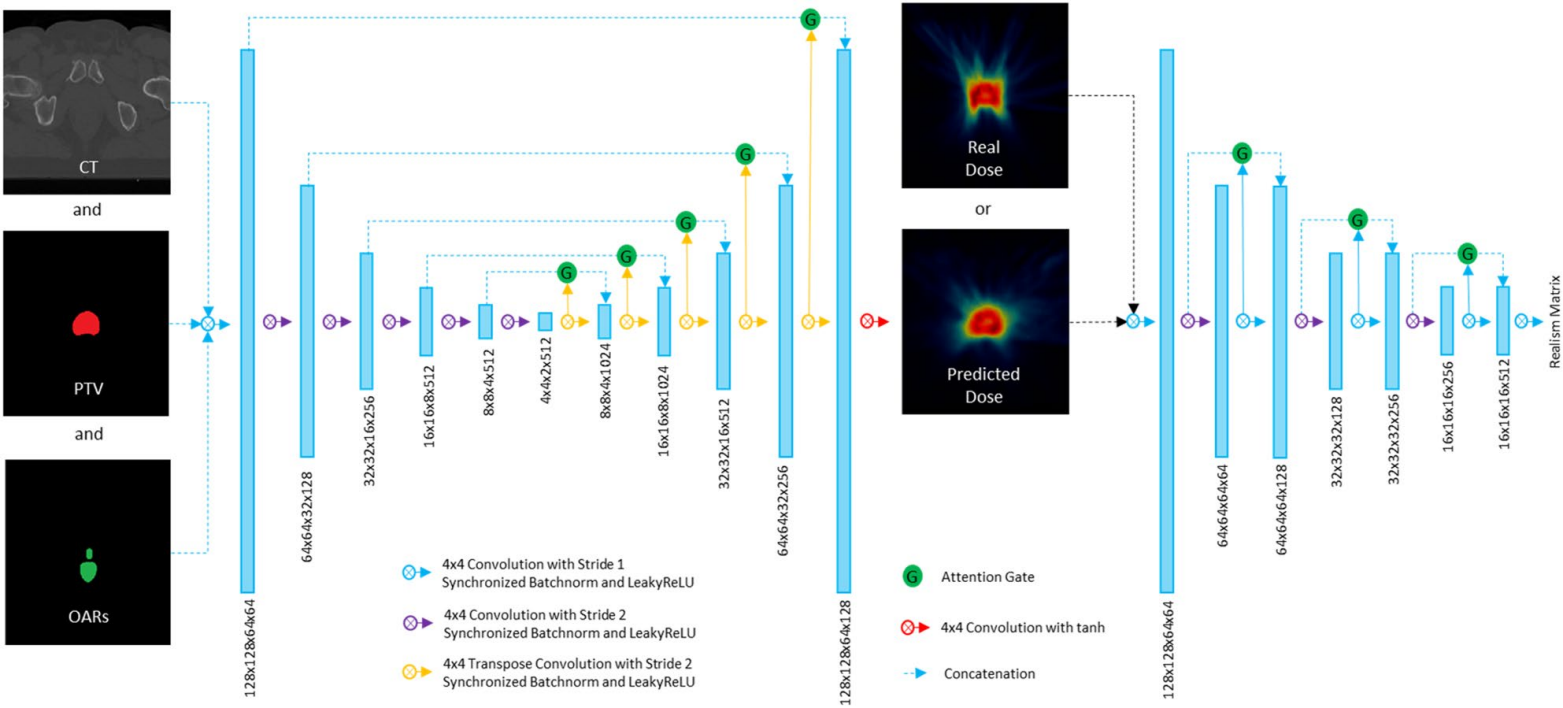
The generator network (left) and discriminator network (right) are shown. The CT, PTV, and OARs are concatenated and fed into the generator network. The discriminator network predicts a realism matrix that attempts to decipher synthetic dose predictions from real dose volumes.

DoseGAN utilizes an attention-aware 3D encoder-decoder variation of the pix2pix generator network^29^. The generator network is five multi-scale levels deep and selectively propagates encoder information directly to the decoder stage through attention gated skip connections. All convolutional layers, except for those residing in the gating mechanism, use 4 × 4 × 4 convolutional kernels with synchronized batchnorm, and leaky ReLU activations. The last layer in the generator network uses hyperbolic tangent activation. The CT, planning target volume (PTV), and organs at risk (OARs) are concatenated and used by the generator network to predict synthetic dose volumes. The predicted synthetic dose and real dose volumes are fed into a densely-connected attention-gated discriminator network which utilizes “PatchGAN” classification to predict a realism matrix that selectively captures local style characteristics^40,41^. The discriminator network is comprised of 8 convolutional layers with 3 convolutional downsampling layers that incrementally reduce the multi-scale resolution of the network. The first layer of each multi-scale level is concatenated to the last layer of each multi-scale level through attention-gated dense-connections. The last convolutional layer of each multi-scale level is used as the gating signal for the attention gated skip connections. Figure 2 shows a schematic of the attention-gated discriminator and generator networks.

### Ground truth

DoseGAN was trained and validated using 126 prostate cancer patients previously treated with SBRT using a CyberKnife (Accuray, Sunnyvale) machine. An additional 15 test patients were used to report final results, following Kaggle-style competition rules. All patients received a monotherapy dose regimen of 38 Gy in 4 fractions, or a 19 Gy boost in 2 fractions and all treatment plans followed peer-reviewed acceptance criteria^42^.

### Training DoseGAN

The discriminator network aims to classify real dose volumes (D Real) as 1 and simultaneously classify predicted dose volumes (D Fake) as 0. DoseGAN uses mean aggregate categorical cross entropy loss from the discriminator and voxel-to-voxel (L1) loss from the generator to update network parameters during training. Introducing L1 loss helps facilitate convergence and enforce spatial congruence in the conditional GAN context.

To avoid multiple hypothesis testing, patients were separated into training, validation, and testing groups, prior to training. In order to mimic the planning environment of the dosimetrist, the model was agnostic to demographic information, and only considered the raw CT image, PTV, OARs, and prescription.

DoseGAN was implemented on a Nvidia V100 graphics processor unit (GPU). Data augmentation was conducted on the fly with the PyTorch data loader using random rigid shifts, rotations, noise, and histogram intensity re-distribution. DoseGAN inferencing took 0.31 s to predict a 128 × 128 × 64 voxel synthetic dose volume and rescale it to its original resolution. The output and input resolutions of DoseGAN were 3 mm  ×  3 mm  ×  3 mm. The data used for this study is not publicly available due to sensitive medical information, but is available from the corresponding author on reasonable request. All patient data has been approved by the Institutional Review Board (IRB) and has been fully anonymized. The methods used in this study were performed in accordance with the University of California San Francisco institutional guidelines. IRB number 14-15452 allowed us to retrospectively collect and analyze our patient dataset. Since this study used retrospective data, informed consent was not required.

### Dosimetric evaluation

DoseGAN was compared to a fully-connected neural network that uses relative distance map information of neighboring input structures (FC), U-Net (UNet), DoseNet, and a 3D GAN architecture (GAN)^18,29,43–45^.

All algorithms were hyperparameter tuned and the model with the best validation performance was saved and used for inferencing on the final test set to report final results. The FC model followed the original model architecture reported in Shiraishi et al., and was trained with 0.45 dropout, a batch size of 4, and a learning rate of 0.01 using Adam optimization^18^. U-Net followed the implementation of the Unet architecture reported in Kearney et al. and was trained with a 0.2 dropout, a batch size of 4, and a learning rate of 0.005 using Adam optimization^21^. DoseNet followed the original implementation reported in Kearney et al. and was trained with a dropout of 0.35, a batch size of 2, and a learning rate of 0.001. For our GAN architecture we used a 3D pix-to-pix implementation by Isola et al. and trained it with a dropout of 0.0, a batch size of 2, and an adaptive learning rate scheduler^26^. It is important to note that we kept the architectures the same or as similar as possible to not detract from their original successful form, however, we conducted a rigorous hyperparameter search to ensure optimal performance on our dataset and a fair comparison. Each algorithm was allowed to max out the memory of the GPU. All models automatically picked the maximum number of parameters before exceeding the memory threshold.

The heterogeneity index (HI), conformity index (CI), and several dose volume objectives were used to evaluate the dosimetric congruence between the synthetic dose predictions and the real ground truth dose. The HI formalism is defined as,

$$HI = \frac{{D_{\max } }}{{D_{p} }}$$,

where D_p_ denotes the prescription and D_max_ denotes the maximum dose value^46^. CI is defined as,

$$CI = \frac{{\left( {TV_{PIV} } \right)^{2} }}{(TV)(PIV)}$$,

where TV is the target volume, TV_PIV_ is the intersection of the target volume and the prescription isodose volume, and PIV is the prescription isodose volume^47^.

DoseGAN predicts the most realistic dose volume given a set of arbitrary input anatomy, as opposed to the best possible dose distribution. Comparator p-values, from a one-sided two-sample Mann–Whitney U test, were used to test if DoseGAN was statistically superior to each alternative dose prediction algorithm. P-values less than 0.05 were considered significant.

## Results

Tables 1 and 2 show the mean values, mean absolute differences between the real dose and each algorithm, and the comparator p-values between DoseGAN and each alternative algorithm. Table 1 shows the PTV V_95_, V_100_, V_120_, and HI for all dose volumes. DoseGAN achieved a statistically significant improvement compared to all alternative algorithms for the V_100_ and V_120_ of the PTV the HI.

**Table 1:** The mean values, mean absolute differences between the real dose and each algorithm, and the comparator p-values between DoseGAN and each alternative algorithm are shown for the V_95_, V_100_, and V_120_ of the PTV as well as the HI.

	Mean values			
	V100-PTV	V95-PTV	V120	HI
Real	97.49 ± 1.06	99.18 ± 0.59	36.88 ± 16.14	1.44 ± 0.12
DoseGAN	97.77 ± 0.77	99.27 ± 0.44	37.5 ± 18.15	1.41 ± 0.1
GAN	97.88 ± 0.67	99.33 ± 0.44	31.86 ± 16.5	1.36 ± 0.11
DoseNet	97.86 ± 0.78	99.23 ± 0.38	30.77 ± 13.23	1.37 ± 0.09
Unet	96.36 ± 1.03	98.88 ± 0.49	20.35 ± 12.89	1.3 ± 0.1
FCN	96.34 ± 1.13	98.48 ± 0.63	29.12 ± 20.21	1.3 ± 0.17

	Mean absolute difference			
	V100-PTV	V95-PTV	V120	HI
DoseGAN	**0.46**	**0.22**	**2.91**	**0.03**
GAN	0.78	0.41	11.4	0.09
DoseNet	0.79	0.35	9.27	0.09
Unet	1.21	0.42	16.54	0.14
FCN	1.17	0.72	12.9	0.15

	P-Values			
	V100-PTV	V95-PTV	V120	HI
GAN	**0.03098481**	0.099253	**0.001845**	**0.003095**
DoseNet	**0.040747324**	0.294869	**0.033998**	**0.013963**
Unet	**0.009033311**	**0.019044**	**5.73E−05**	**0.003508**
FCN	**0.028196182**	**0.012551**	**2.4E−05**	**0.001073**

Bold font indicates the least difference between the real and predicted dose and statistically significant p-values.

**Table 2:** The mean values, mean absolute differences between the real dose and each algorithm, and the comparator p-values between DoseGAN and each alternative algorithm are shown for the CI, V_60_ of the bladder, V_60_ of the rectum, and mean dose of the bulb.

	Mean values			
	CI	V60-Bladder	V60-Rectum	Mean-bulb
Real	0.81 ± 0.07	5.71 ± 3.68	6.59 ± 3.93	4.13 ± 2.78
DoseGAN	0.79 ± 0.06	6.42 ± 4.06	8.13 ± 4.79	4.79 ± 2.93
GAN	0.79 ± 0.06	7.25 ± 4.35	4.1 ± 3.85	5.04 ± 3.09
DoseNet	0.8 ± 0.06	8.64 ± 3.76	5.88 ± 4.37	5.13 ± 2.49
Unet	0.77 ± 0.07	7.99 ± 3.83	9.15 ± 4.12	3.94 ± 2.88
FCN	0.74 ± 0.1	6.51 ± 4.93	6.55 ± 6.85	3.08 ± 2.53

	Mean absolute difference			
	CI	V60-Bladder	V60-Rectum	Mean-bulb
DoseGAN	**0.03**	**1.55**	**1.67**	**1.16**
GAN	0.04	3.06	3.19	1.76
DoseNet	0.05	3.36	2.71	1.43
Unet	0.06	3.04	3.36	1.66
FCN	0.08	3.44	3.24	1.65

	P-Values			
	CI	V60-Bladder	V60-Rectum	Mean-bulb
GAN	0.131377	**0.033998**	**0.023243**	**0.040747**
DoseNet	**0.033998**	0.073288	**0.037248**	0.565887
Unet	**0.02562**	**0.028196**	**0.019044**	0.073288
FCN	**0.02562**	**0.015508**	**0.033998**	0.370011

Bold font indicates the least difference between the real and predicted dose and statistically significant p-values.

Table 2 shows the CI, V_60_ of the bladder, V_60_ of the rectum, and mean dose of the penile bulb for all dose volumes. DoseGAN achieved a statistically significant improvement compared to all alternative algorithms for the V_60_ of the rectum.

**Figure 3:**
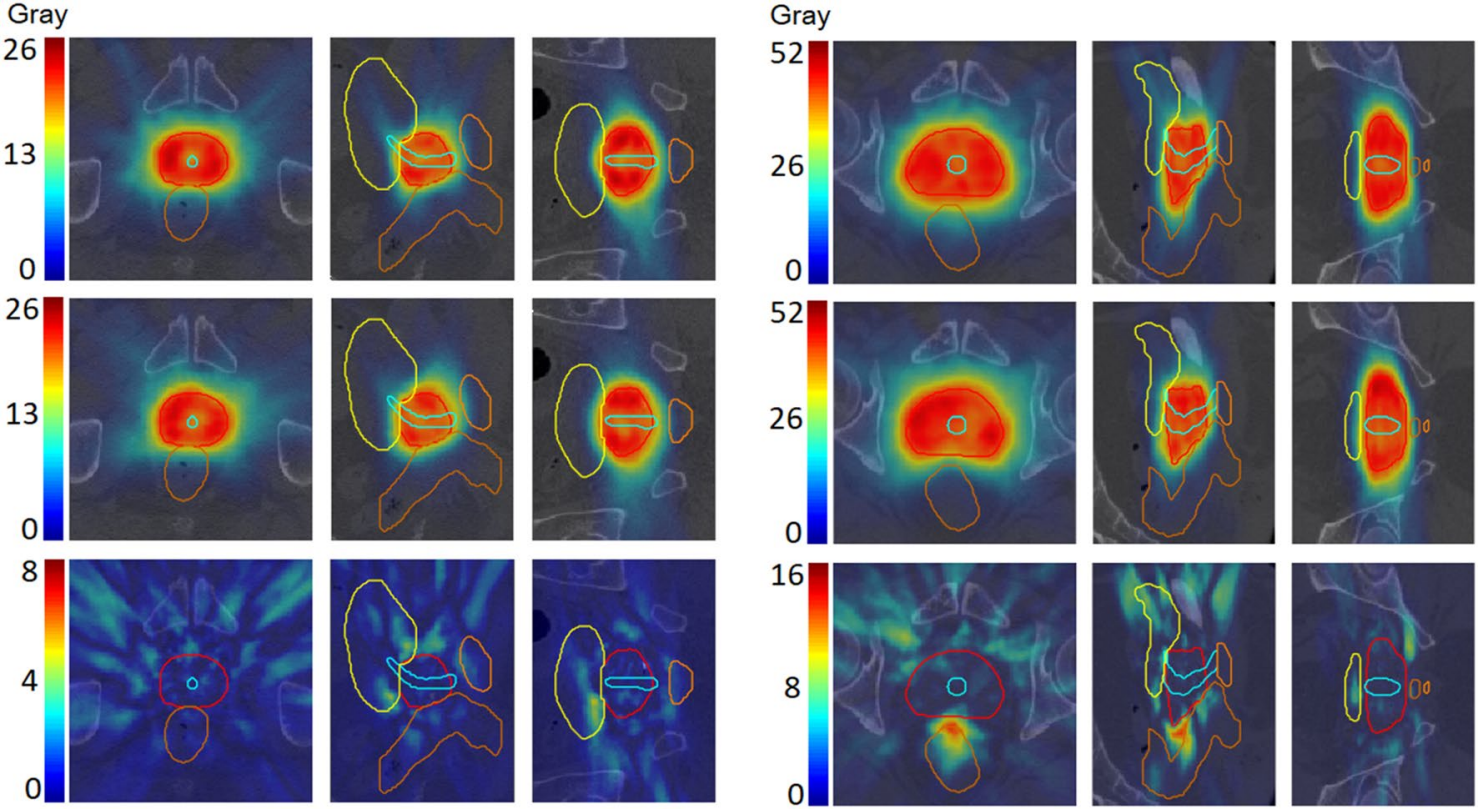
The original real dose (top), DoseGAN synthetic dose (middle), and dose difference (bottom) are shown for patients 7 (left) and 20 (right). The PTV, rectum, bladder, and penile bulb are shown in the red, brown, yellow, and orange contours, respectively. Axial, sagittal and coronal slices are shown from left to right.

Figure 3 shows the real dose, DoseGAN predicted synthetic dose, and dose difference for two patients. DoseGAN was able to achieve realistic synthetic dose predictions compared to the original real plans, as seen in Fig. 3.

**Figure 4:**
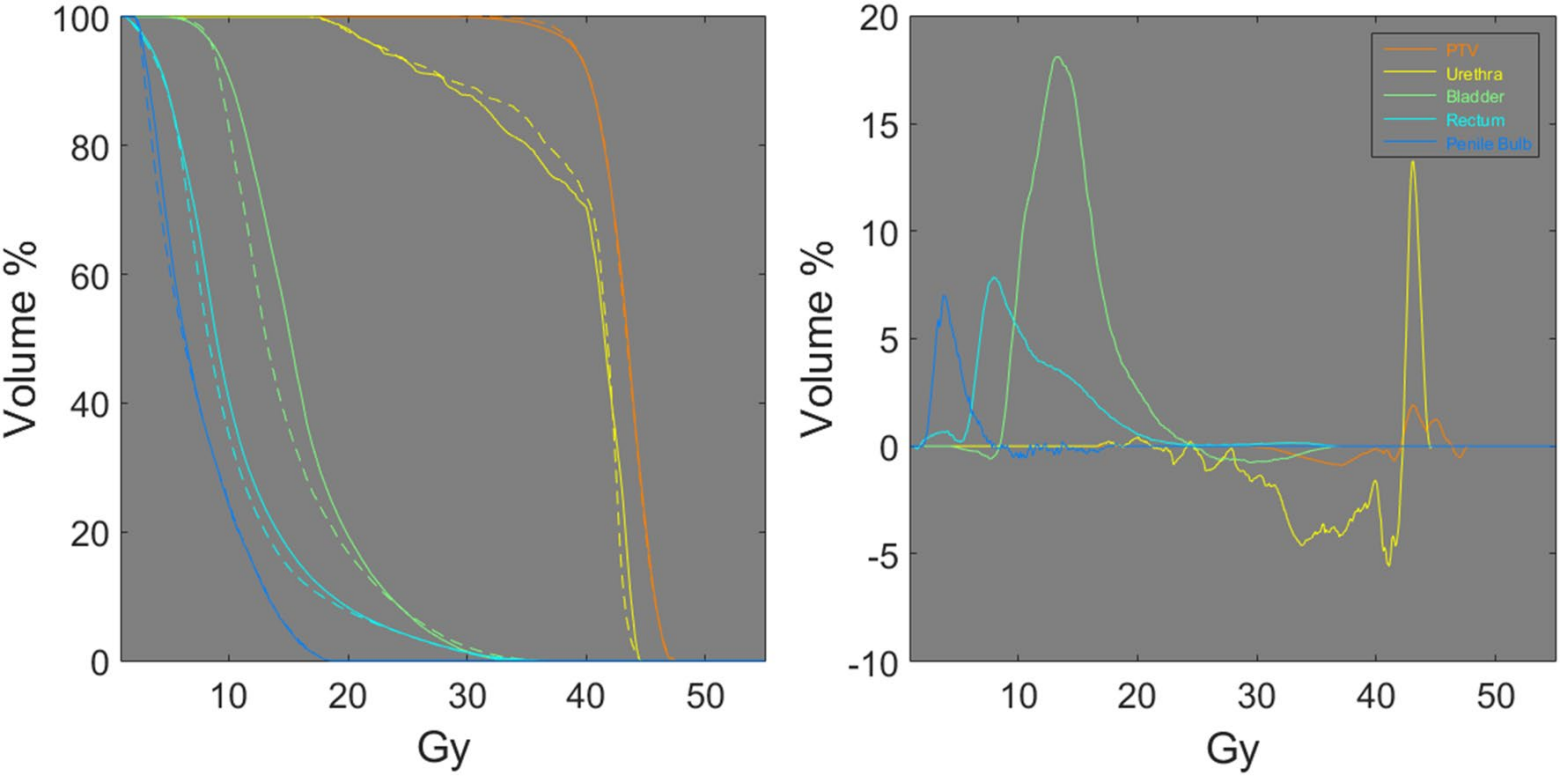
The real DVHs (solid line) (left), DoseGAN DVHs (dashed line) (left) and the DVH differences (right) are shown for a 38 Gy plan. The PTV, urethra, bladder, rectum, and penile bulb are shown in orange, yellow, green, teal, and blue respectively.

Figure 4 shows the dose volume histograms (DVHs) and DVH differences between the real dose distributions and DoseGAN synthetic dose distributions for the PTV, urethra, bladder, rectum, and penile bulb for 38 Gy plan. DVHs represent the radiation dose to tissue volume and the DVH differences represent the difference between the planned DVH of the predicted DVH.

**Figure 5:**
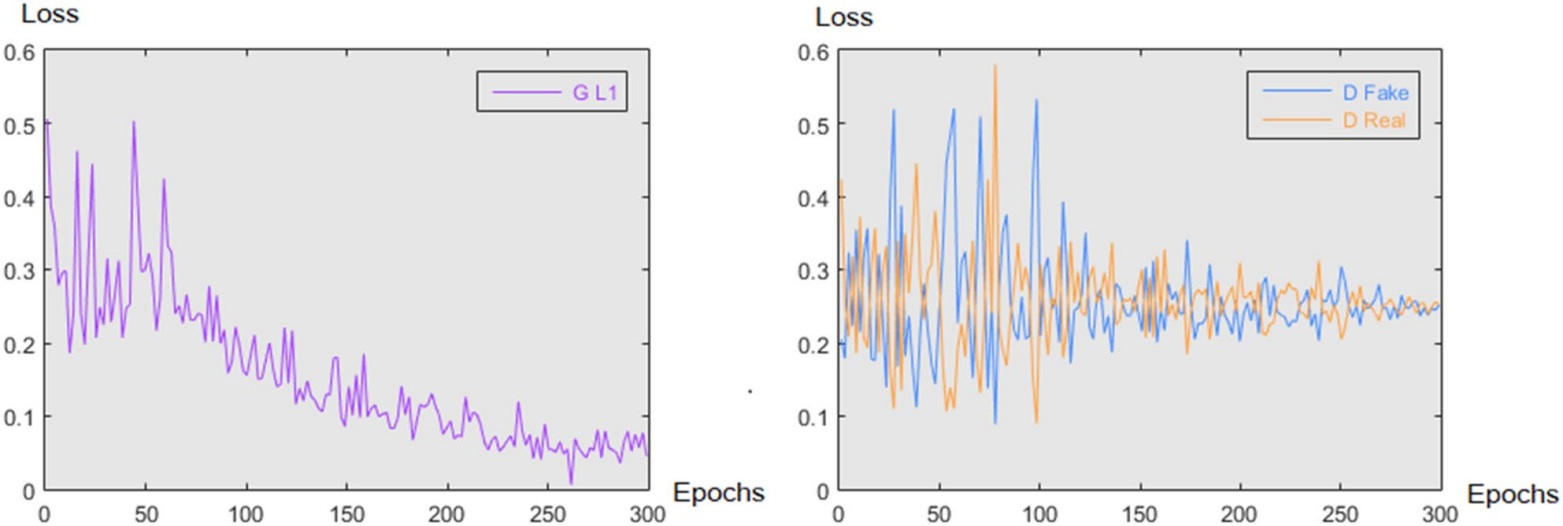
The L1 loss from the generator (left) is shown in purple and the D Fake and D real losses from the discriminator (right) are shown in blue and orange respectively for all epochs during training for the DoseGAN model.

Figure 5 depicts the loss at each epoch for the DoseGAN algorithm. The L1 loss from the generator and the discriminator losses can be seen progressing in unison during model training.

## Discussion

This study demonstrates the superiority of a novel conditional generative adversarial attention-gated network for SBRT synthetic dose prediction. This is the first ever implementation of generative adversarial attention-gated networks to this problem space.

On average DoseGAN was able achieve more realistic dose predictions compared to all other algorithms by learning a realism matrix that helped mimic the dosimetric nuances of real clinical SBRT plans. DoseGAN achieved statistically significant improvement compared to all alternative algorithms for the V_100_ and V_120_ of the PTV, HI, and V_60_ of the rectum.

The conventional GAN algorithm achieved good results for the V_95_ of the PTV, CI, and V_60_ of the bladder, but did not perform as well as DoseGAN for the V_100_ and V_120_ of the PTV, and V_60_ of the rectum. Similarly, DoseNet achieved good results for the V_95_ of the PTV, mean dose of the penile bulb, and V_60_ of the bladder, but did not perform as well as DoseGAN for the V_100_ and V_120_ of the PTV, HI, V_60_ of the rectum, and mean dose of the penile bulb.

Table 1 shows that DoseGAN performs much better than the alternative algorithms for the target V_120_ and HI. While conventionally fractionated dose regimens tend to have much smoother dose distributions, SBRT plans tend to have intentional hotspots within the main tumor volume. The alternative algorithms consistently predicted lower target V_120_ and HI values, meaning that the plans have less dose escalation within the target volume and implying a loss in clinical efficacy.

Table 2 shows that DoseGAN performed better at predicting the dose to the V_60_ of the rectum and V_60_ of the bladder, which is partially due to the stochastic nature of SBRT plans. Pure spatial loss algorithms failed to model the hot or cold spots within the sensitive organs. All algorithms performed well for the mean bulb since this metric takes the average dose to the structure and is more forgiving than structures that are more sensitive to hot spots. All algorithms also performed well for the CI, since the CI is a measurement of the target coverage and our dataset of dose volumes were fairly consistent with regards to this metric.

The models with pure spatial loss tended to produce overly smooth synthetic dose distributions and were not able to capture the heterogeneous hotspots and cold spots that are endemic to SBRT dose volumes. Pure spatial loss, such as mean squared error between the dose volumes, will produce the most likely dose at each voxel given a set of inputs. However, in the presence of dose heterogeneity or inconsistent planner preferences, conventional CNNs will learn to predict a best approximation of the dose in order to reconcile the inconsistent dose targets with respect to the input variables. Since conventional CNNs reach a compromise with respect to varied learning objectives, they are inherently disadvantaged compared to architectures that do not rely on pure spatial loss, such as GANs.

Since GANs are difficult to train, the number of network parameters needs to be kept as low as possible to facilitate adversarial training. Attention gates were used to reduce redundancy within the network, improve efficiency, and facilitate model convergence, which enabled a deeper discriminator architecture. The realism matrix was able to incorporate broader dosimetric information, since it uses a deeper discriminator which allows for a wider receptive field.

The model architecture of all algorithms, such as the depth, number of filters at each layer, and other hyperparameters, were determined using the validation set and were designed to stay within the memory limitations of the GPU hardware used in this study. Since GANs are notoriously difficult to train, DoseGAN borrowed many architectural design elements form the original pix2pix network, such as the size of each convolutional kernel, and relative location and type of various network activations.

This study has some limitations. Since this study was only conducted on SBRT prostate patients, it is not clear if this approach would work non-SBRT plans. Also, DoseGAN was trained to predict dose volumes with a 3 × 3 × 3 mm^3^ voxel resolution. Although this resolution is clinically acceptable, typical SBRT dose calculations tend to use 1 × 1 × 1 m^3^ or 2 × 2 × 2 mm^3^ voxel resolutions. Increasing the resolution of DoseGAN would increase the number of parameters, change the receptive field of the model, and require more GPU memory. More extensive hyperparameter tuning and greater hardware resources would also be necessary to determine the viability of finer resolution dose prediction. Also, the number of parameters for each model was restricted by the GPU memory since only one GPU was used in this study. Also, the number of parameters is not the only determining factor in memory allocation. Each intermediary output layer is held in GPU memory, so networks that have more layers at higher resolutions will be more memory intensive. Hyperparameter tuning assured a balance between memory utilization at the upper multi-scale levels and lower-multi levels. Since the hyperparameter tuning stage automatically picked the upper memory limit for each model, we can assume that each model would have achieved better results with a bigger batch size and more parameters^48^. Furthermore, DoseGAN was only evaluated on abdominal anatomy, so it can not be assumed that DoseGAN will work on other anatomical regions.

In spite of these limitations, dose prediction using attention-aware generative adversarial networks presents a viable solution to dose prediction for prostate SBRT patients. Clinically incorporating DoseGAN would help conserve hospital resources by determining achievable plan dosimetry at the time of CT simulation as opposed to after the entire treatment planning process. Furthermore, DoseGAN could be used as a clinical decision support tool or be incorporated into the plan optimization process, to help improve plan quality and reduce the strain on clinical resources.

## Conclusions

We have developed a novel attention-aware generative adversarial network for synthetic dose prediction that was able to achieve superior dose prediction accuracy compared to current alternative state-of-the-art methods. DoseGAN presents a solution to overcome the challenges of realistic volumetric dose prediction in the presence of diverse patient anatomy.
